# Gender Differences in Commuting Injuries in Spain and Their Impact on Injury Prevention

**DOI:** 10.1155/2017/3834827

**Published:** 2017-11-26

**Authors:** Miguel A. Camino López, Óscar J. González Alcántara, Ignacio Fontaneda

**Affiliations:** Department of Civil Engineering, University of Burgos, Burgos, Spain

## Abstract

A gender analysis of workers injured while commuting in Spain is presented, distinguishing between injury due to traffic-related accidents and injury due to other causes.* Method*. A total of 266,646 traffic-related injuries and 168,129 nontraffic-related injuries are studied over the period 2006–2010.* Results*. In Spain, the accident rate recorded in working hours is much higher among men; nevertheless, it is curious that commuting-related accident rates are higher among women than men, in both traffic-related injuries and nontraffic-related injuries. The study of the frequency distribution confirmed that many more injuries occurred in Spain while commuting to work rather than from work and that women suffered twice as many injuries as men at nine in the morning. Musculoskeletal disorders are the only injuries that registered a higher number of cases among women and falls to the same level are the most relevant cause among women.* Conclusions*. The analysis of these and more findings established that a great effort should go into the promotion of preventive measures in favour of women workers. These results may encourage companies to modify their accident prevention plans, so as to increase their effectiveness in the struggle against occupational accidents following the five points described in this article.

## 1. Introduction

In the European Union, the number of women employees has been increasing, following a trend that is moving towards equality with men. In fact, the gap between male and female employment rates has fallen by 15.5 points [1]. In Spain, it is also moving towards greater equality, such that, in 2008, the average working population among women increased by 2.0%, while it fell among men by 2.2% [2]. In 2011, a drop in the working population took place in both groups, although that reduction was greater among men (−2.9%) than among women (−0.7%) [3].

These trends have prompted one of the most extensive debates currently underway in the scientific literature: to establish whether gender is a risk factor in occupational accident rates. In particular, do men suffer more injuries at work than women or do women have more injuries? Various studies on heavy industry in the USA [4] and in the health sector of the Canadian province of British Columbia [5] have arrived at the conclusion that it is women who have a greater probability of suffering occupational injuries. It has also been demonstrated that women have a greater risk of suffering musculoskeletal disorders that may not be attributed solely to excessive reporting of injuries [6] or due to an increased stress at workplace [7]. A Directorate General of the European Commission, named EUROSTAT, provides statistical information to the institutions of the European Union; it establishes a type of injury in Code 030 named “Dislocations, Sprains, and Strains” as “all acute musculoskeletal problems due to overexertion of muscles, tendons, ligaments, and joints" [8]. This code is similar to the one collected as Code 3 of the International Labor Organization.

On the contrary, other activities, such as electricity supply companies in Southern California [9] and Construction in the USA [10], record much higher injury rates among men than among women. Moreover, studies in countries as different as Sweden [11] and Taiwan [12] have also reached the same conclusion.

Other studies have analyzed the causes seeking to explain these differences in occupational injuries among men and women. In doing so, various authors have analyzed different physical capabilities and double presence [1]:

1. *Different physical capabilities* between men and women in combination with workplace design are frequently cited as key factors in women's occupational health. Studies that have measured physical strength among men and women report that women have 30 to 70% less upper body strength than men and 5 to 20% less lower body strength [9]. Different physical capabilities could be one reason for the different injuries between men and women. Thus, it has been confirmed that women have a higher accident rate than men in chronic musculoskeletal disorders [13], in general and among grocery workers [14]. Likewise, the occurrence of slips, trips, and falls in the working day among women was 50% greater than those registered among men [15]. If we look at occupations such as driving a taxi, a greater prevalence of injuries to the neck may be seen among injured Norwegian women, both women taxi drivers (70.8%) and the reference population (57.6%). The most frequently injured area for men is the lumbar region, both among taxi drivers (58.5%) and the reference population (50.7%) [16].
2. *Double presence [17]*: it appears that women workers spend a lot of their free time on unremunerated domestic chores and on the care of children and the elderly. When the hours worked in the main occupation and in secondary jobs, plus the time invested in displacements and in domestic chores, are added up, it is confirmed that women work longer hours than men. This disparity points to a very clear illustration of the “double role” that women play in the job market and in the home [1, 18]. In addition, that greater level of dedication to the home and to the care of children and elderly people means that women need family conciliation more and that their stress levels are higher than those of men [9].

On the other hand, traffic-related injuries have a decisive influence on occupational injury rates. In fact, the seriousness of an injury is affected to a great extent by consequences of a traffic crash in the course of the working day or on the way to or from work. A total of 23% of all deaths in the United States in 2012, related to occupational situations, were due to traffic crashes [19, 20]. There was 30% in Canada [21], 60% in France [22], 49% in Australia [23], and 50% in Finland [24].

Gender differences have also been observed in traffic crash injuries. In this way, several researches demonstrate that male gender is a factor for road accidents, including those related to work [25]. In addition, it has been confirmed too with the excessive confidence in the male group of a younger age; in fact, despite perceiving the risks when driving [26], these young drivers tend to believe that the risk does not affect them. These risks are often merely taken for fun and a total of 31% of young men admitted taking risks for this reason as opposed to 7% of women [27]. In addition, various studies on driving have pointed out that men, especially young men, show higher risk behaviours and attitudes than women [28–30].

Commuting accidents are the injuries that workers suffer on the way to or from work. In the European Union there are an estimated 3,400 fatal and 650,000 nonfatal accidents that result in over three days' absence from the workplace [31].

These injuries are not recognized as occupational accidents in all countries of the European Union. In fact, from among the 27 countries of the EU, all commuting accidents are only registered as such in Austria, Germany, Belgium, Spain, Finland, French, Holland, Italy, Luxembourg, Poland, Portugal, Romania, Slovenia, and Sweden [32].

Commuting accidents in Spain are considered occupational accidents. An “occupational accident” is defined as “the injury suffered by the worker as a consequence of the work that is undertaken” by Article 115 of the RDL 8/2015 [33]. Therefore, occupational injury and workplace accident are therefore similar concepts. Occupational diseases are not sudden actions but delayed in time; they are caused by chemical, physical, biological, and carcinogenic agents and by inhalation of substances and skin diseases [34].

Among commuting accidents, we can distinguish between traffic-related and nontraffic-related injuries. Among the former, we distinguish between when travelling in a vehicle and when on foot. Among the latter, the vast majority occur when travelling on foot.

More men than women drive at all ages, so they are likely to be pedestrians less frequently. In this sense, the most obvious explanation of the greater risk of injury for women is that they drive less, probably walk more, so they will be more exposed to risk situations as pedestrians [35]. Moreover, the stretch covered on foot is the most dangerous part of the trip to work [24].

Despite these studies, it remains unclear whether gender is, as many researchers suppose, a risk factor. In this study, we seek to contribute to this debate, through a statistical analysis of accidents in the course of commuting in Spain over a five-year period (2006–2010), comparing gender aspects, both for injuries that are traffic-related and that are nontraffic-related.

## 2. Materials and Methods

In Spain, companies are obliged to insure their employees and to notify the Ministry of Employment and Social Security of all injuries to their employees in the workplace that involve one day or more away from work. Moreover, taking into account the fact that the daily compensation rate paid to a worker is notably higher when it is due to a workplace injury, we may consider that accident notifications in this country are around 100%.

Finally, the duty doctor has the obligation of diagnosing the severity of occupational injuries. A total of 97.9% of the commuting injuries under analysis were categorized as slight, 1.8% were diagnosed as serious, and, finally, 0.3% had fatal consequences.

As we know, workers suffer what are known as commuting injuries when travelling to and from work. Part of these occupational accident rates are traffic-related: for example, the traffic injury that the work suffers when going to work, either in a vehicle as a passenger or driver or as a pedestrian hit by a car. Another part of the commuting accidents are nontraffic-related, for example, the worker that injures a foot stepping off the curb or going down the stairs at home on the way to work.

### 2.1. Data Collection

All commuting-related injuries of one day or more off work in Spain, over the period 2006–2010, were selected for our study. The data on injuries were taken from the existing notifications held on file at the Spanish Ministry of Employment and Social Security. The number of injuries under analysis amounted to four-hundred and thirty-four thousand, seven hundred and seventy-five (434,775) of which 50.9% involved men and 49.1% women. Those days off work that were a consequence of relapses due to injuries caused by occupational accidents suffered in the past were excluded.

The injury rate was calculated per 1,000 workers, dividing the number of injuries by the number of workers that were exposed in any one year. In Spain, it is estimated that a maximum of 1.5% of the total number of workers carried out their work at home [36]. In view of the impossibility of estimating the percentage of men and in women, it was decided to consider the same number of workers for the calculation of the incident rates while commuting and in working hours. The data on employment for the denominators of these rates were also taken from the Spanish Ministry of Employment and Social Security.

The serious and mortal injury rates were calculated by dividing the number of serious and fatal injuries by the number of workers. This denominator is the same as that used for the other rates that were calculated, for ease of understanding, per 100,000 workers.

### 2.2. Study Design

Our aim is to establish the differences in the gender of workers that suffer commuting accidents. To do so, the accident rate trends of men and women in the workplace and while commuting were studied. In addition, gender differences while commuting were analyzed on the way to and from work, both for traffic-related and for nontraffic-related injuries. Finally, the differences in gender were studied through the adjustment of commuting accidents by different risk factors, such as age and time of the accident.

It was decided to study commuting accidents exclusively with the purpose of avoiding any possible vertical and horizontal segregation suffered at work among women.

### 2.3. Statistical Analysis

The studies that correlated the gender of the injured person and the other variables were done with contingency tables, in which the value of the Chi-Squared statistic (*χ*^2^) was calculated, to test the hypothesis of independence of the variables under analysis with regard to gender. This statistic will manifest the possible influence of these variables on the gender of the injured person.

The corrected standardized residuals (c.s.r.) were also obtained from the contingency tables, in which an asterisk appears next to those with a value below 1.96 in absolute terms. These values do not therefore reach a statistical significance of 95%, which would be sufficient to reject the hypothesis of independence of the variables. We can affirm that the influence is more than random for those values that are higher than 1.96 in absolute terms.

All the analyses were done using the SPSS V18 statistical software package.

## 3. Results

Table 1 shows us the number of men and women workers in each of the productive sectors in Spain in 2010. It appears to confirm a horizontal segregation by gender, as for every 100 workers in the construction sector, 11.6 are women and, in contrast, for every 100 workers in the services sector, 54.1 are women.

### 3.1. Injury Ratios

**Figure 1:**
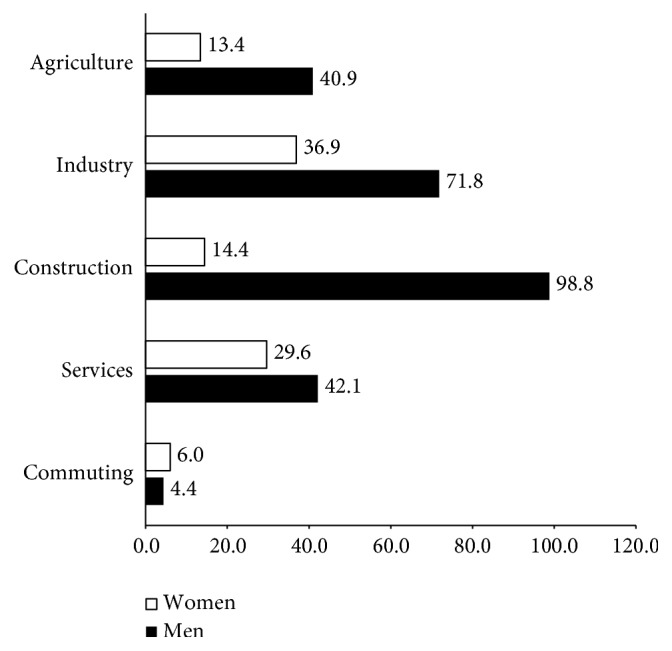
Injury rates by gender (injury × 1,000 workers). Spain 2010.* Source*. Prepared by the authors using the anonymous data on occupational accidents provided by the Subdirección General de Estadística of the Ministerio de Empleo y Seguridad Social.

**Figure 2:**
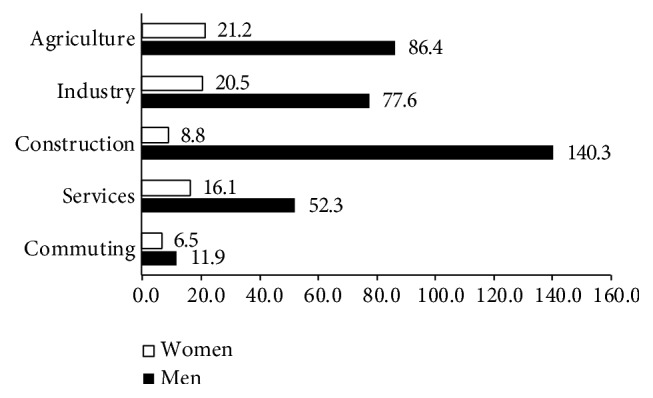
Injury rates for both serious and mortal injuries (injury × 1,000 workers). Spain 2010.* Source*. Prepared by the authors using the anonymous data on occupational accidents provided by the Spanish Subdirección General de Estadística of the Ministerio de Empleo y Seguridad Social.

In Figure 1, it may be seen that, in all of the production sectors, the incidence rate is higher among men than among women. These differences could be due to vertical segregation; in other words, men and women develop different tasks and occupy different posts, although they work in the same sector. This conclusion would appear to be confirmed in Figure 2, which shows that the serious and fatal injury ratios are much higher among men than among women, in all productive sectors.

Curiously, the incident rate is inverted for commuting accidents, becoming greater among women (6.0) than among men (4.4). This fact repeated itself in all the years under analysis. Thus, in Table 2, it may be seen that the incident rate among men was more than double (except for 2009) that registered among women for injuries during the working day. In contrast, the incident rate for the five years studied for commuting accidents was always higher among women.

In the European Union, the incidence rate of commuting accidents varies somewhat between sectors of economic activity. The incidence rate is slightly higher for women than for men, in most sectors, especially the construction, transport, and telecommunications sectors [31].

Analyzing the incident rate in Spain for commuting accidents by sectors for the year 2010, it is confirmed that in agriculture (men: 1.6, women: 1.0) and construction (men: 4.5, women: 3.9) the ratio was higher among men than among women; however, both in industry (men: 4.2, women: 5.5) and in services (men: 4.8, women: 6.6) the ratio was greater among women. However, despite the higher incident rate for commuting accidents among women, the likelihood of suffering a serious or fatal injury in commuting was greater among men than among women.

**Figure 3:**
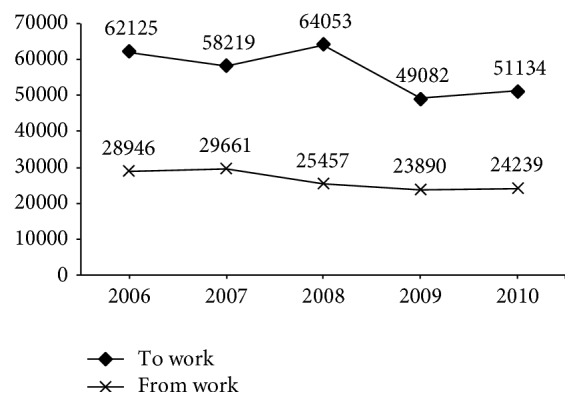
Total injuries due to commuting accidents while travelling to and from work. Spain 2006–2010.* Source*. Prepared by the authors using the anonymous data on occupational accidents provided by the Spanish Subdirección General de Estadística of the Ministerio de Empleo y Seguridad Social.

Moreover, in Figure 3, it was confirmed that commuting accidents occurred with greater frequency when travelling to work rather than from work. In fact, more than twice as many accidents occurred when travelling to rather than from work.

Calculation of the incident ratios of men and women, for the year 2010, when travelling to and from work, confirmed that, logically, both groups registered higher rates when travelling to work than from work. However, although the ratio among men was 2.9 while travelling to work and 1.5 from work, the ratio among women was 4.2 and 1.8, respectively.

The injury ratios were subsequently calculated for traffic-related commuting accidents and nontraffic-related accidents. We know that workers can suffer traffic-related accidents when travelling to work, as either drivers, passengers, or pedestrians. Equally, they can suffer a nontraffic-related injury, for example, on the stairs when leaving their home. The total number of workers, both men and women, was therefore used as the divisor, for the calculation of the ratios for both for traffic-related and nontraffic-related injuries.

In Table 3, we check that women have had higher incident rates in commuting accidents over recent years than men. So, with regard to nontraffic-related commuting accidents, women have for all years studied had higher rates than their male colleagues.

### 3.2. Traffic-Related and Nontraffic-Related Injuries

Accident rates by gender are not always easy to calculate, due to not knowing the number of men and women that work weekends or that enter or leave the workplace at a particular time. Therefore, the frequency distributions of the injuries are analyzed, both for traffic-related and nontraffic-related injuries.

First of all, we confirmed the importance of commuting among women; thus, while women suffered 23.6% of injuries in the working day, they registered 49.1% of all commuting accidents.

As shown in Table 4, commuting accidents have progressively fallen from 2007. The economic crisis has influenced this fall, causing an unusual rise in unemployment that has affected Spain in an especially intense way. However, this reduction has not occurred with equal intensity among both genders. Table 4 shows us that injuries in commuting accidents suffered by women increased every year and nontraffic-related injuries were higher among women year after year.

**Figure 4:**
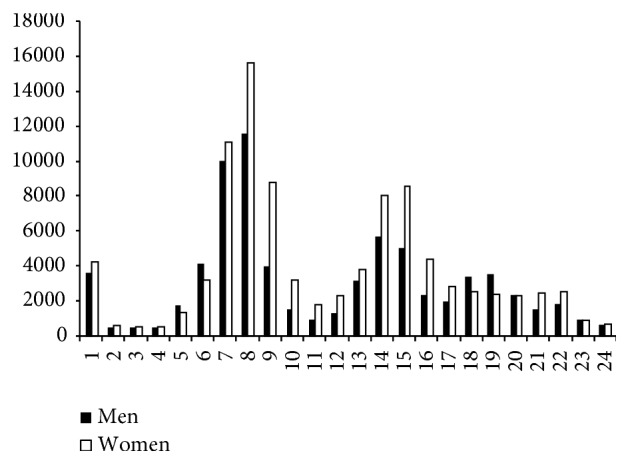
Nontraffic-related commuting accidents by time of day and by gender. Spain 2006–2010.* Source*. Prepared by the authors using the anonymous data on occupational accidents provided by the Spanish Subdirección General de Estadística of the Ministerio de Empleo y Seguridad Social.

**Figure 5:**
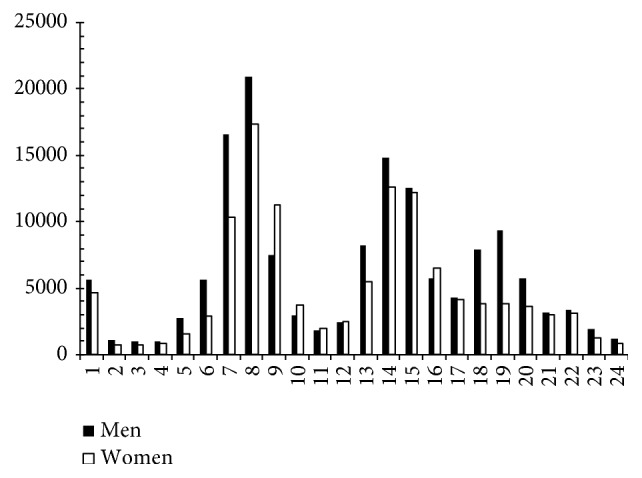
Traffic-related commuting accidents by time of day and by gender. Spain 2006–2010.* Source*. Prepared by the authors using the anonymous data on occupational accidents provided by the Spanish Subdirección General de Estadística of the Ministerio de Empleo y Seguridad Social.

In Figures 4 and 5, we can see that, for traffic-related and for nontraffic-related injuries, the number of injuries suffered by women at 09:00 hours is very much higher than those suffered by men at 09:00 hours.

Adjusting nontraffic-related commuting accidents by time and by gender (*χ*^2^ = 3,804.668; d.f.: 23; *p* < 0.001), we can see that the greatest differences between men and women were registered for injuries at nine a.m., of which men suffered 4,043 injuries and women 8,754 injuries (c.s.r.: 28.8). With regard to traffic-related injuries (*χ*^2^ = 6144.472; d.f.: 23; *p* < 0.001), there were 7,513 cases among men and 11,213 (c.s.r.: 43.9) among women.

### 3.3. Age and Seriousness

The highest number of commuting accidents was recorded for both men and women in the 26–35 age range. As is shown in Table 5, the trend followed by women in nontraffic-related injuries may be underlined, as their percentages increased considerably with age.

In contrast, greater seriousness in the injuries suffered by men and fatalities may be seen in Table 6, for both traffic-related and nontraffic-related injuries.

### 3.4. Type of Contract

From among every 100 injuries due to commuting accidents among men, 6.4% of those injured held temporary contracts. Among women the percentage rose to 22.1%.

We can see from Table 7 that these differences apply to both traffic-related and nontraffic-related accidents.

### 3.5. Type of Injury and Injury by Body Part

**Figure 6:**
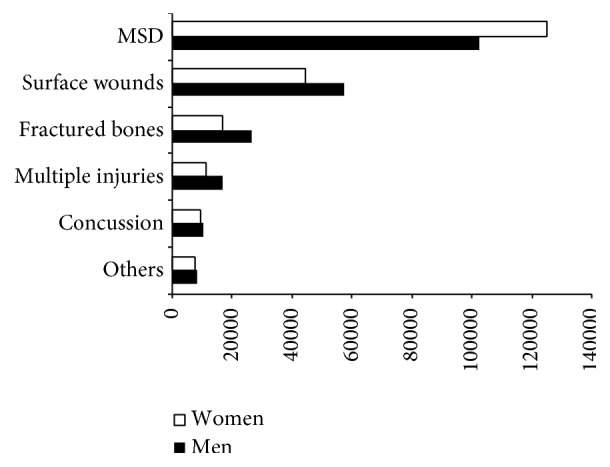
Injuries in commuting accidents by gender. Spain 2006–2010.* Source*. Prepared by the authors using the anonymous data on occupational accidents provided by the Spanish Subdirección General de Estadística of the Ministerio de Empleo y Seguridad Social.

The most frequent injuries among both men and women are Musculoskeletal Disorders (MSD) (52.3%) (see Figure 6) as it is established in Code 030 of EUROSTAT methodology [8]. Adjusted by gender (*χ*^2^ = 7,181.776; d.f.: 5; *p* < 0.001), for every 100 injuries of this type, women suffered 54.9% (c.s.r.: 80.6) and men suffered 45.1% (c.s.r.: −80.6).

Analyzing injuries in traffic-related accidents while commuting by gender (*χ*^2^ = 9210.332; d.f.: 5; *p* < 0.001), 50.5% of these injuries resulted in MSD. Likewise, for every 100 injuries of this type, women suffered 52.5% (c.s.r.: 84.3) and men suffered 47.5% (c.s.r.: −84.3). In contrast, fractures represented 8.2% of all injuries and men suffered 76.1% (c.s.r.: 63.8), as opposed to the 23.9% (c.s.r.: −63.8) suffered by women.

With regard to the injuries suffered in nontraffic-related commuting accidents (*χ*^2^ = 390.963; d.f.: 5; *p* < 0.001), MSD represented 55.0% of these injuries, of which women suffered 58.3% (c.s.r.: 18.8). Fractures represented 12.6% of these accidents, of which women suffered 54.3% (c.s.r.: −6.4).

**Figure 7:**
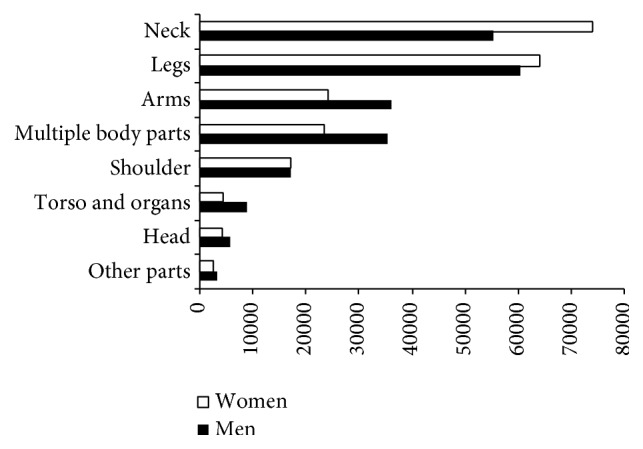
Part of the body injured in commuting accidents by gender. Spain 2006–2010.* Source*. Prepared by the authors using the anonymous data on occupational accidents provided by the Spanish Subdirección General de Estadística of the Ministerio de Empleo y Seguridad Social.

In Figure 7, we see that the most frequently injured area of the body was the neck, in 29.7% of the total, which affected women in 73,825 cases. However, the highest number of cases among men was registered in the legs with a total of 60,395 cases.

Table 8 confirms that injuries to the neck are prominent in traffic-related injuries among women. With regard to nontraffic-related injuries, injuries to the neck only represent 7.3% of the total and there are no statistically significant differences by gender. A greater number of injuries was registered among both men and women, in the legs.

### 3.6. Physical Activity and Cause of the Injury

The physical activity undertaken by the worker at the time of suffering the injury is analyzed in this section. The main physical activities are

1. driving or travelling in a means of transportation: 63.7%,
2. entering, leaving, walking, running, and jumping: 30.9%.

Finally, 5.4% of injured workers were conducting other physical activities when the injury was inflicted.

Within the first activity (1), we can distinguish between injuries: (1) while driving a means of motorized transport; (2) while driving a nonmotorized means of transport; (3) while travelling as a passenger; and (4) others.

Table 9 highlights the distribution of injuries that are inflicted by activity (1), highlighting the percentages obtained by women in the section “(3) Passenger.”

**Figure 8:**
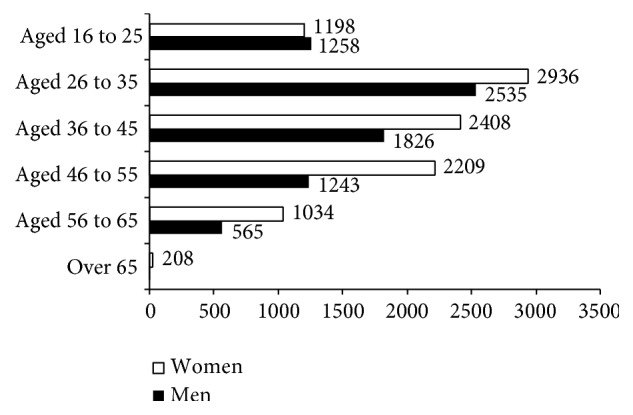
Injuries going up and down stairways by age and by gender. Spain 2006–2010.* Source*. Prepared by the authors using the anonymous data on occupational accidents provided by the Spanish Subdirección General de Estadística of the Ministerio de Empleo y Seguridad Social.

We now move on to analyze the injuries suffered by men and women when going up and down stairways in buildings. From among a total of 17,240 injuries, women suffered 56.9%, registering important differences in accordance with age, as may be seen in Figure 8.

**Figure 9:**
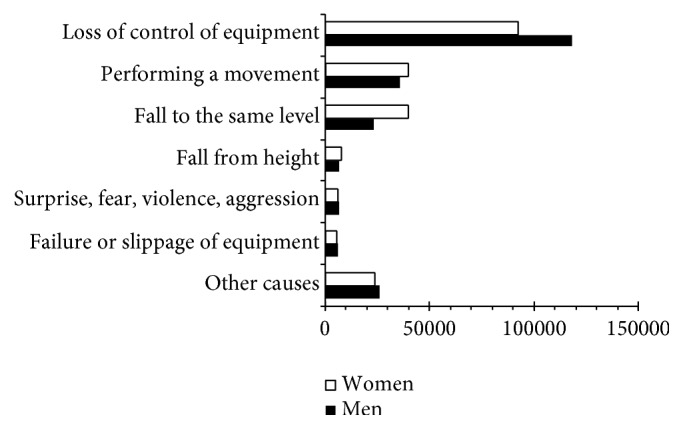
Injuries by cause and by gender. Spain 2006–2010.* Source*. Prepared by the authors using the anonymous data on occupational accidents provided by the Spanish Subdirección General de Estadística of the Ministerio de Empleo y Seguridad Social.

The cause of the injury in commuting accidents also presented important differences by gender as is seen in Figure 9.

Almost 80% of the traffic-related injuries (*χ*^2^ = 818.714; d.f.: 6; *p* < 0.001) had two direct causes:

1. Loss of control of equipment or vehicles
2. Performing body movements (the following activities are recorded in this section: stepping on an object, kneeling down, crouching, sitting down, turning, transporting something and pushing or depositing an object)

In the first case, women suffered 82,931 injuries (44.4%; c.s.r.: −1.4) and in the second 12,583 (50.3%; c.s.r.: 19.3).

The following causes are highlighted with regard to the nontraffic-related injuries (*χ*^2^ = 5,520.535; d.f.: 6; *p* < 0.001):

1. Falls at the same level: 55,718
2. Performing body movements: 50,255
3. Falls at a different level: 10,725

Women suffered 66.6% (c.s.r.: 59.8) of falls to the same level, 53.8% (c.s.r.: −13.2) of which were due to bodily movements and 61.1% (c.s.r.: 10.3) to falls to a different level.

## 4. Discussion

Every year, the number of women workers is greater in both Spain and the European Union. Studies are therefore necessary that will provide insight into whether gender is a risk factor in injuries suffered in occupational accidents.

Improvements to workplace organization and the moderation of physical effort in jobs mean that women are occupying jobs that were traditionally occupied by men [9], although significant horizontal segregation persists as shown by their significantly lower representation that continues in jobs such as construction, mining, the wood industry, and the iron and steel industry [11, 37]. This important horizontal segregation was also detected in our study; in fact, the representation of women in the workforce is very different in each productive sector. Thus, it was 11.6% in the construction sector, 24.8% in industry, 41.6% in agriculture, and 45.1% in the services sector.

The probability of suffering an injury is always greater among men than among women, but this difference is very significant in some sectors. Thus, the incident rate, in 2010, of men in the agrarian sector was three times higher than women and almost eight times higher in the construction sector. Even though women continue to take up jobs that were traditionally done by men, it appears that the high-risk jobs continue to be done by men.

One way of trying to remove the effects of vertical and horizontal segregation consists in analyzing the injuries suffered in commuting accidents as men and women, in principle, should perform the same jobs and tasks, which consist in going to work, whether as a pedestrian, a passenger, or a driver. In fact, while women suffered 23.6% of injuries that occurred in the working day, they registered 49.1% of the commuting accidents.

In this type of injury, there is a radical change in tendency. Women register a far greater probability of suffering an injury while commuting. However, this trend only holds true for the industry and services sector, as men registered a higher incidence rate in the agricultural and the construction sectors. This fact should be the object of further investigation.

Moreover, it appears that women workers dedicate more time than men to the care of their children and the elderly; in other words, they play a “double role” [1, 18]. Analyzing the injuries at the time they occur, we find that, in the first place, nine in the morning is the time of day with the greatest differences between the injuries suffered by men and by women. In fact, at that time, women suffered 1.5 times more traffic-related commuting injuries than men and 2.2 times more in nontraffic-related commuting accidents. Considering that the normal school opening hours in Spain are between nine and ten in the morning, the presence of women at that time would be twice as much.

Moreover, special attention should be given to the journey to work, as both men and women registered a higher incident rate on their way to rather than from work.

The percentages of serious injuries among women were higher on the way to work (24.1%) than on the way home (21.8%). It is possible that the increased stress that the “double presence” causes among women [9] might influence these percentages.

Besides, the number of injuries has fallen in recent years. This fall may be due to the economic crisis that Spain has suffered with special intensity. However, the percentage of both traffic-related and nontraffic-related injuries suffered by women is greater each year, such that, in 2006, women suffered 40.4% of all traffic-related injuries and 50.5% of all nontraffic-related injuries. In 2010, those percentages rose to 48.8% and 61.9%, respectively.

Younger people, aged between 16 and 25 years, suffered over 20% of traffic-related injuries. From among these, 58.8% were men and 41.2% were women. The seriousness of the injury was much greater among men than among women, although no significant differences were registered among the youngest workers. The youngest workers do not appear to run greater risks than their companions of an older age when travelling to work.

With regard to nontraffic-related injuries, the percentages of accidents among women increased with age, moving from 44.6% in the 16- to 25-year-old range, to 66.9% in the 56- to 65-year-old range.

Women occupied 73% of part-time jobs in the European Union [1]. In our study, the importance of part-time contracts appears to be relevant in injuries among women. In fact, from among the commuting injuries, 14.1% were suffered by people with a part-time contract and, of these, 76.8% were women, while 44.0% of those with a full-time contract were women.

Various authors have confirmed that women suffer more MSD [6] in the working day [13]. Women registered a greater percentage of MSD than men in both the traffic-related and the nontraffic-related commuting accidents, as well as those due to other causes. These findings appear to confirm the greater probability of this type of injury among women.

With regard to the injured body part, in Copenhagen, female office workers suffered a higher percentage of severe neck/shoulder pain than male [38]. Women also suffered a higher number of neck injuries than men, in traffic-related injuries, as was established for Norwegian taxi drivers [16].

Moreover, approximately 95% of the people that suffered an injury while commuting was carrying out one of the following activities: (1) driving or travelling as a passenger in a vehicle (63.7%) and (2) entering, leaving, walking, and running (30.9%). In the former activity, women suffered 43.5% of all injuries. However, the high percentage suffered by women when travelling as passengers is significant (51.4%).

Even more significant is that 61% of the injuries occurring in the second activity were to women. This result is complemented with the analysis of the causes of injury in nontraffic-related commuting accidents, which found that women suffered 66.6% of all falls at the same level and 61.1% of falls at a different level. As happened in the injuries in the working day [15], women appear to suffer a higher number of commuting injuries due to falls at the same level than men. Finally, an analysis of the injuries that men and women have suffered going up and down stairways shows that the age of the women is an important risk factor. In fact, women suffered 48.8% of accidents on stairways among the 16- to 24-year-old age group, while they suffered 64% among the 45- to 54-year-old age group.

## 5. Conclusions: Impact on Injury Prevention

The conclusions of this article may be used by companies to improve the results of their accident prevention programmes. The main contributions may be summarised under the following five points:

1. The probability of suffering an injury during the working day is greater among men in all of the productive sectors. Vertical and horizontal gender segregation may justify this fact. However, the probability of suffering an injury in commuting accidents is greater among women, in both traffic-related and nontraffic-related injuries.
2. The probability of suffering an injury in a commuting accident is much greater when travelling to work than from work. We considered that the same number of workers travelled to and from home to their place of work.
3. Family-oriented activities should be organized to try to reduce the number of injuries due to commuting accidents that women suffer at nine a.m., the time school usually opens in Spain.
4. It is important that women are made aware of the risks, above all, in traffic-related injuries, of neck injuries.
5. Preventive efforts are necessary to reduce the significant number of injuries due to falls at the same and at different levels suffered by women. High-heeled shoes often worn by women might provide some explanation for that high accident rate.

We believe that greater effort should go into the promotion of preventive measures in favour of women workers, with a view to avoiding the high number of injuries in commuting accidents among women in comparison to their male colleagues.

## Figures and Tables

**Table 1:** Distribution of workforce by gender and productive sector. Spain 2010.

Productive sectors	Men	Women
Agriculture	633,222	452,073
Industry	1,555,506	512,327
Construction	1,039,554	136,907
Services	4,762,558	5,620,727

*Source*. Data provided by the Subdirección General de Estadística of the Ministerio de Empleo  y Seguridad Social.

**Table 2:** Incident rate by gender (injury × 1000 workers).

	Working hours		Commuting	
	Male	Female	Male	Female
2006	80.6	29.5	5.6	6.5
2007	78.2	29.7	5.7	6.6
2008	67.2	27.6	5.0	6.4
2009	55.7	24.6	4.6	6.2
2010	50.8	22.7	4.4	6.0

*Source*. Prepared by the authors using the anonymous data on occupational accidents provided by the Subdirección General de Estadística of the Ministerio de Empleo y Seguridad Social.

**Table 3:** Commuting injury rates by gender and by traffic (injury × 1000 workers).

	Traffic-related		Nontraffic-related	
	Male	Female	Male	Female
2006	3.3	3.2	2.3	3.3
2007	3.9	3.8	1.7	2.8
2008	3.5	3.8	1.5	2.6
2009	3.2	3.5	1.5	2.7
2010	3.0	3.4	1.4	2.7

*Source*. Prepared by the authors using the anonymous data on occupational accidents provided by the Subdirección General de Estadística of the Ministerio de Empleo y Seguridad Social.

**Table 4:** Frequency and percentage distribution of injuries in commuting accidents by gender and by year. Spain (2006–2010).

	Traffic-related injury				Nontraffic-related injury			
	Number	Men (%)	Women (%)	c.s.r.^*∗∗*^	Number	Men (%)	Women (%)	c.s.r.^*∗∗*^
*Total*	*266,646*	*55.5*	*44.5*		*168,129*	*43.7*	*56.3*	
	Chi-Squared = 1,158.572 d.f. = 4			*p* < 0.001	Chi-Squared = 1,224.170 d.f. = 4			*p* < 0.001
2006	50,580	59.6	40.4	−20,7	42,122	49.5	50.5	−27,5
2007	62,251	58.6	41.4	−17,8	34,986	46.1	53.9	−9,9
2008	57,778	54.8	45.2	3,7	31,780	42.3	57.7	5,8
2009	49,806	52.2	47.8	16,4	30,097	39.9	60.1	14,8
2010	46,231	51.2	48.8	20,4	29,144	38.1	61.9	21,2

*Notes*. ^*∗∗*^The csr refers to the column “percentages of accidents suffered by women.” *Source*. Prepared by the authors using the anonymous data on occupational accidents provided by the Subdirección General de Estadística of the Ministerio de Empleo y Seguridad Social.

**Table 5:** Frequency and percentage distribution of commuting accidents by gender and by age. Spain (2006–2010).

	Traffic-related injuries				Nontraffic-related injuries			
	Number	Men (%)	Women (%)	c.s.r.^*∗∗*^	Number	Men (%)	Women (%)	c.s.r.^*∗∗*^

*Total*	*266,645*	*55.5*	*44.5*		*168,129*	*43.7*	*56.3*	
	Chi-Squared = 1,212.053 d.f. = 5			*p* < 0.001	Chi-Squared = 3,657.171 d.f. = 4			*p* < 0.001
From 16 to 25 years	54,422	58.8	41.2	−17.5	21,813	55.4	44.6	−37.2
From 26 to 35 years	106,102	51.8	48.2	31.5	47,853	48.0	52.0	−22.4
From 36 to 45 years	63,155	56.7	43.3	−6.9	40,514	45.6	54.4	−8.6
From 46 to 55 years	31,950	57.0	43.0	−5.9	37,281	35.3	64.7	37.0
From 56 to 65 years	10,829	63.7	36.3	−17.7	20,206	33.1	66.9	32.5
Over 65 years	187	62.0	38.0	−1.8^*∗*^	462	27.5	72.5	7.0

*Notes*. ^*∗*^Corrected standardized residuals < 1.96 in absolute value. ^*∗∗*^The csr refers to the column “percentages of accidents suffered by women.” *Source*. Prepared by the authors using the anonymous data on occupational accidents provided by the Subdirección General de Estadística of the Ministerio de Empleo y Seguridad Social.

**Table 6:** Frequency and percentage distribution of commuting accidents by gender and by seriousness. Spain (2006–2010).

	Traffic-related injuries				Nontraffic-related injuries			
	Number	Men (%)	Women (%)	c.s.r.^*∗∗*^	Number	Men (%)	Women (%)	c.s.r.^*∗∗*^
*Total*	*266,646*	*55.5*	*44.5*		*168,129*	*43.7*	*56.3*	
	Chi-Squared = 1,400.883 d.f. = 2			*p* < 0.001	Chi-Squared = 257.270 d.f. = 2			*p* < 0.001
Slight	259,357	54.9	45.1	37.2	166,210	43.5	56.5	13.1
Serious	6,142	75.8	24.2	−32.4	1,731	54.9	45.1	−9.5
Fatal	1,147	82.5	17.5	−18.4	188	90.4	9.6	−12.9

*Notes*. ^*∗∗*^The csr refers to the column “percentages of accidents suffered by women.” *Source*. Prepared by the authors using the anonymous data on occupational accidents provided by the Subdirección General de Estadística of the Ministerio de Empleo y Seguridad Social.

**Table 7:** Frequency and percentage distribution of commuting accidents by gender and by type of contract. Spain (2006–2010).

	Traffic-related injury				Nontraffic-related injury			
	Number	Men (%)	Women (%)	c.s.r.^*∗∗*^	Number	Men (%)	Women (%)	c.s.r.^*∗∗*^
*Total*	*265,749*	*55.5*	*44.5*		*167,337*	*43.8*	*56.2*	
	Chi-Squared = 13,483.003 d.f. = 2			*p* < 0.001	Chi-Squared = 9,535.525 d.f. = 2			*p* < 0.001
Full-time contract	226,759	60.1	39.9	−115.4	137,241	49.3	50.7	−97.3
Part-time temporary contract	34,542	27.5	72.5	112.2	26,676	17.7	82.3	93.7
Part-time permanent contract	4,448	37.7	62.3	24.1	3,420	25.6	74.4	21.7

*Notes*. ^*∗∗*^The csr refers to the column “percentages of accidents suffered by women.” A “part-time permanent contract” refers to an employment contract for services that are not required on a continuous basis every day of the year. *Source*. Prepared by the authors using the anonymous data on occupational accidents provided by the Subdirección General de Estadística of the Ministerio de Empleo y Seguridad Social.

**Table 8:** Frequency and percentage distribution of injuries in commuting accidents by gender and injured area. Spain (2006–2010).

	Traffic-related injuries				Nontraffic-related injuries			
	Number	Men (%)	Women (%)	c.s.r.^*∗∗*^	Number	Men (%)	Women (%)	c.s.r. ^*∗∗*^
*Total*	*266,646*	*55.5*	*44.5*		*168,129*	*43.7*	*56.3*	
	Chi-Squared = 17,218.156 d.f. = 7			*p* < 0.001	Chi-Squared = 1,134.503 d.f. = 7			*p* < 0.001
Neck	116,735	42.8	57.2	116.7	12,283	43.0	57.0	1.7^*∗*^
Legs	33,061	70.4	29.6	−58.3	91,279	40.7	59.3	27.5
Arms	26,827	74.3	25.7	−65.5	33,174	48.3	51.7	−18.8
Multiple body parts	47,387	64.0	36.0	−41.2	11,328	43.8	56.2	−0.1^*∗*^
Shoulders	24,438	50.5	49.5	16.4	9,571	47.8	52.2	−8.3
Torso and organs	8,885	70.9	29.1	−29.8	4,403	57.8	42.2	−19.1
Head	5,309	65.0	35.0	−14.1	4,472	50.2	49.8	−8.8
Other parts	4,004	59.6	40.4	−5.3	1,619	46.7	53.3	−2.4

*Notes*. ^*∗*^Corrected standardized residuals < 1.96 in absolute value. ^*∗∗*^The csr refers to the column “percentages of accidents suffered by women.” *Source*. Prepared by the authors using the anonymous data on occupational accidents provided by the Subdirección General de Estadística of the Ministerio de Empleo y Seguridad Social.

**Table 9:** Frequency and percentage distribution of injuries by gender driving or on board a means of transport. Spain (2006–2010).

	Number	Men (%)	Women (%)	c.s.r.^*∗∗*^
*Total*	*276,938*	*56.5*	*43.5*	
	Chi-Squared = 1,279.757 d.f. = 4			*p* < 0.001
Not specified	19,575	60.5	39.5	−11.7
(a) Driving a motorized vehicle	208,500	56.5	43.5	0.1^*∗*^
(b) Driving a nonmotorized vehicle	15,276	64.7	35.3	−21.1
(c) Passenger	27,874	48.6	51.4	28.1
(d) Other activities	5,713	59.5	40.5	−4.7

*Notes*. ^*∗*^Corrected standardized residuals < 1.96 in absolute value. ^*∗∗*^The csr refers to the column “percentages of accidents suffered by women.” *Source*. Prepared by the authors using the anonymous data on occupational accidents provided by the Subdirección General de Estadística of the Ministerio de Empleo y Seguridad Social.
